# Genotype: A Crucial but Not Unique Factor Affecting the Clinical Phenotypes in Fabry Disease

**DOI:** 10.1371/journal.pone.0161330

**Published:** 2016-08-25

**Authors:** Xiaoxia Pan, Yan Ouyang, Zhaohui Wang, Hong Ren, Pingyan Shen, Weiming Wang, Yaowen Xu, Liyan Ni, Xialian Yu, Xiaonong Chen, Wen Zhang, Li Yang, Xiao Li, Jing Xu, Nan Chen

**Affiliations:** The department of Nephrology, Ruijin Hospital, the Medical School affiliated to Shanghai Jiaotong University, Shanghai, China; Cincinnati Children's Hospital Medical Center, UNITED STATES

## Abstract

Numerous α-galactosidase A (α-gal A) gene (GLA) mutations have been identified in Fabry disease (FD), but studies on genotype-phenotype correlation are limited. This study evaluated the features of GLA gene mutations and genotype-phenotype relationship in Chinese FD patients. Gene sequencing results, demographic information, clinical history, and laboratory findings were collected from 73 Chinese FD patients. Totally 47 mutations were identified, including 23 novel mutations which might be pathogenic. For male patients, those with frameshift and nonsense mutations presented the classical FD, whereas those with missense mutations presented both of classical and atypical phenotypes. Interestingly, two male patients with missense mutation p.R356G from two unrelated families, and two with p.R301Q from one family presented different phenotypes. A statistically significant association was found between the levels of α-gal A enzyme activity and ocular changes in males, though no significant association was found between residual enzyme activity level and genotype or clinical phenotypes. For female patients, six out of seven with frameshift mutations and one out of nine with missense mutation presented the classical FD, and α-gal A activity in those patients was found to be significantly lower than that of patients with atypical phenotypes (13.73 *vs*. 46.32 nmol/ml/h/mg). Our findings suggest that the α-gal A activity might be associated with the clinical severity in female patients with FD. But no obvious associations between activity level of α-gal A and genotype or clinical phenotypes were found for male patients.

## Introduction

Fabry disease (FD, MIM 301500), an X-linked lysosomal storage disorder, is caused by deficient activity of the α-galactosidase A enzyme (α-gal A, E.C.3.2.1.22) [1]. The enzyme deficiency may lead to a progressive intracellular accumulation of complex sphingolipids, particularly globotriaosylceramide (Gb3). The accumulation of these products is the basis of FD symptomology that includes acroparesthesias, angiokeratomas, corneal and lenticular opacities, gastrointestinal problems, as well as renal, cardiac and cerebrovascular diseases [1].

The α-galactosidase A is encoded by GLA gene (12 kb) which is located on the X chromosome (Xq22) and contains seven exons (92 to 291 bp) [2, 3]. More than 600 mutations of GLA gene have been reported in FD patients, but a few of them have been identified in Chinese patients [4, 5]. Recently, increasing attention has been focused on assessing the association between genotype and phenotype in FD patients. Since 2002, our department has followed 71 Fabry families, which comprises the largest Fabry family group in China. The clinical and pathological manifestations and laboratory findings of all patients were collected, and GLA gene mutations were surveyed. Our aim was to define the features of GLA gene mutations and the genotype-phenotype relationship in Chinese FD patients.

## Materials and Methods

### Patients and controls

#### Inclusion criteria

The FD was diagnosed in accordance with the UK guidelines (2005): characteristic ultrastructural findings (myeloid body) and/or deficient activity of α-gal A in plasma or leukocytes. If the histological or enzyme activity were not assessed, the diagnosis of FD was made according to the following two criteria: (1) clinical manifestations including acroparesthesia, angiokeratoma, characteristic corneal opacities and/or tortuosity of the conjunctival and retinal vessels, proteinuria and/or kidney failure, cardiomyopathy and cerebrovascular abnormalities such as transient ischemic attacks, strokes; (2) family history of acroparesthesia, cardiomyopathy, renal disease or stroke.

DNA/RNA samples obtained from 73 FD patients belonging to 58 unrelated Chinese families were sequenced. DNA samples obtained from 70 unrelated healthy persons (30 women and 40 men) were selected as normal controls. Demographic information, clinical and family history, and laboratory findings were collected from all subjects.

All procedures in the present study were performed in accordance with the ethical standards of the responsible committee on human experimentation (institutional and national) and with the Helsinki Declaration of 1975, as revised in 2000. The ethics committee of Ruijin Hospital approved the study. Informed written consent was obtained from all participants.

### Mutation analysis

#### Conventional genomic sequencing

Genomic DNA was extracted from peripheral blood samples with a GenElute blood genomic DNA kit (Sigma-Aldrich, St. Louis, MO, USA) according to the manufacturer’s instructions, or from hair follicles using the method as described by Higuchi R *et al* [6, 7]. Six pairs of oligonucleotide primers (primer sequences were not shown but available on request) were designed to amplify the seven exons and flanking intronic regions of the GLA gene (NG_007119.1). PCR products were purified and directly sequenced using an ABI 3700 automated DNA sequencer (Perkin-Elmer Applied Biosystems, Foster City, CA, USA).

#### RT-PCR sequencing

Total RNA was isolated from peripheral neutrophils using TRIzol (Invitrogen, New York, USA). Real-time PCR was performed with a Roche Lightcycler and Qiagen QuantiTect One Step RTPCR SYBR green kit (Qiagen, Germany) following manufacturer’s instructions. Two overlapping PCR fragments were amplified and sequenced using two pairs of primers (available on request) designed according to the GLA gene (NM_000169).

#### Sequence analysis

Sequence analysis and mutation identification were performed by using Sequencing Analysis software (Perkin-Elmer Applied Biosystems). For novel missense mutations, SIFT (Sorting Intolerant From Tolerant, http://sift.jcvi.org/) and Poly-Phen (http://genetics.bwh.harvard.edu/pph2/) were used to assess pathogenic possibilities.

### Activity measurement of α-gal A

The α-gal A activity in leukocytes was detected using the fluorimetric method described by Desnick *et al* [8]. The normal range of α-gal A activity is defined as higher than 37.0 nmol/ml/h/mg in our laboratory.

### The definition of clinical phenotypes

Clinical phenotype of FD was divided into two groups: classical FD and atypical FD [9–13]. Then, atypical FD was subdivided into renal-dominant FD and cardiac-dominant FD (S1 Table).

Patients were grouped as classical FD if they met the following criteria: 1) The activity of α-gal A in leucocytes was less than 5% of the reference value (37 nmol/ml/hr/mg) for male patients or less than the reference value for female patients; 2) Onset age was less than 25-years old independent of gender; 3) patient had a GLA gene variation, and suffered from at least one of the typical FD symptoms (neuropathic pain, cornea verticillata and clustered angiokeratoma) or had one or more family members with a definite diagnosis of classical FD.

Atypical FD was defined as the presence of an FD-like sign or symptom not specific to FD, such as left ventricular hypertrophy (LVH). Biopsies of affected organ were conducted to confirm the diagnosis if the storage pattern was demonstrated to be characteristic for FD on Electron Microscopy (EM). Biopsies were also carried out in family members carrying the same GLA variant. As to atypical FD, the criteria for classification were independent of gender. Specifically, patients with predominantly renal involvement were classified as renal-dominant FD, patients with predominantly cardiac involvement were classified as cardiac-dominant FD.

The definition of kidney involvement was as follows: eGFR was calculated using the Chronic Kidney Disease Epidemiology Collaboration (CKD-EPI) equation [14]. Chronic kidney disease (CKD) was classified based to the Kidney Disease Outcomes Quality Initiative (K/DOQI) practice guidelines [15]. Proteinuria was defined as 24-hour urinary protein excretion >150mg. Renal function deterioration was defined as EPI-eGFR < 90ml/min. Occurrence of end stage renal disease (ESRD) was defined as eGFR<15 ml/min/1.73m^2^ and the need for renal replacement therapy (dialysis or renal transplantation).

### Statistical analysis

Data were expressed as median (range). Mann-Whitney U test was used to evaluate the associations of α-gal A activity with genotype, clinical phenotypes and clinical manifestations of different organs. A *P*-value <0.05 was considered to be significantly different. Statistical analysis was conducted by using SPSS ver. 19.0 software (SPSS Software, Chicago, IL, USA).

## Results

### Mutations identified in the GLA gene

**Fig 1:**
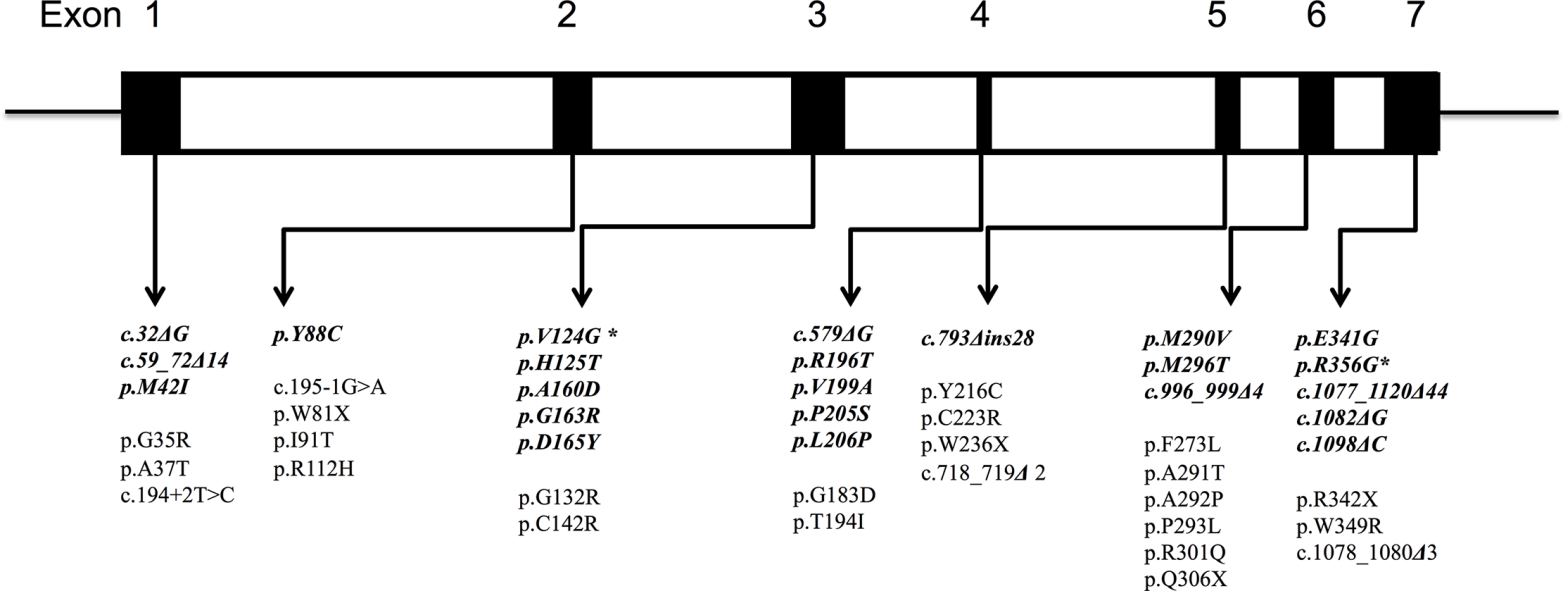
Schematic of the α-galactosidase A gene indicating the relative position of the seven exons and showing the 47 mutations, including 23 novel mutations (Bold Italic). Abbreviations used: P205S (proline to serine substitution at codon 205); ins, insertion; Δ, deletion. Numbers for deletions or insertions refer to the nucleotide position in the α-galactosidase A cDNA sequence. *novel mutation found in two presumably unrelated families.

Totally, 47 mutations detected in 58 families were considered to be pathogenic, among which 31 (66.0%) were missense mutations, 8.5% were nonsense mutations, and 4.3% were splicing mutations (Fig 1). Twenty-three of these mutations had not been reported previously, including 15 missense mutations (p.M42I, p.Y88C, p.V124G, p.H125T, p.A160D, p.G163R, p.D165Y, p.R196T, p.V199A, p.P205S, p.L206P, p.M290V, p.M296T, p.E341G, p.R356G), 5 small deletions (c.32delG, c.579delG, c.996_999delACAG, c.1082delG, c.1098DelC), two large deletions (c.59_72DelCCCTCGTTTCCTGG, c.1077_1120del44bp) and one complex rearrangement mutation (c.793_809delins28bp).

Four novel small deletions (not c.996_999delACAG) and two novel large deletions resulted in a frameshift and early termination. Two single-base deletions (c.1082delG and c.1098DelC) predicted a premature termination in codon 391. A 14-base deletion (c.59_72DelCCCTCGTTTCCTGG) introduced a frameshift in codon 20, altering residues 21–29 and then introduced a termination signal in codon 30. A 44-base deletion resulted in substitution of isoleucine for lysine at codon 359, followed by a premature termination in codon 379. A four-base deletion (c.996_999delACAG) was detected in the female proband from F65, which had no available RNA sample. This mutation predicted the following possible products: one resulted in the alternation in codon 332 and introduced a premature termination in codon 345, the other might affect the signal splice; the final product could not be predicted.

One complex rearrangement mutation was identified in a male proband from family F44. In this case, 17-base pairs (c.793_809) in exon 5 were deleted and substituted by another 28 bp, which changed the residues from codon 265 and resulted in a prolonged protein containing 455 amino acids.

A reported splice-site variation (c.195-1G>A in intron 1) [16] was identified in a male patient from family F10. Our reverse transcriptase-PCR demonstrated that this splicing mutation resulted in seven-base deletion in exon 2, leading to an early termination of α-gal A at codon 118.

### Phenotype and genotype relationship

The clinical data of 73 patients (56 males, 17 females) from 58 families carrying GLA mutations were shown in Tables 1–3. Thirty-nine patients underwent renal biopsy and one patient underwent nerve biopsy (Tables 1–3). In all renal specimens, myeloid bodies were clustered in the podocyte cytoplasm. For some classical FD patients, myeloid bodies were also found in mesangial cells and renal tubular epithelial cells (S1 Fig). Myeloid bodies were also found in nerve cells.

**Table 1:** Mutations and major clinic data of patients with classic FD.

	Mutations	Nucleotide change	Novel	Gender	Age at onset/ diagnosis	Clinical phenotypes	Family history	Angiokeratoma	Acroparesthesias	Hypohidrosis	Ocularchanges^$^	Hearing loss	Proteinuria	Baseline eGFR (EPI)	LVH	Hypertension	Cerebrovascular abnormalities^&^	Myeloid body (EM)	α-gal A activity (nmol/ml/h/mg)
	**Missense**																		
**F06-A**	p.G132R	c.394G>C	**N**	**M**	**4/20**	**classic**	**pos**	**Y**	**Y**	**Y**	**Y**	**Y**	**Y**	**13.11**	**Y**	**Y**	**Y**	**Y^**	**0.25**
**F12-A**	p.G183D	c.548G>A	**N**	**M**	**10/21**	**classic**	**pos**	**Y**	**Y**	**Y**	**Y**	**Y**	**Y**	**48.22**	**N**	**Y**	**Y**	**Y**	**0.43**
**F69-A**	p.T194I	c.581C>T	**N**	**M**	**12/25**	**classic**	**pos**	**Y**	**Y**	**Y**	**Y**	**N**	**Y**	**125.71**	**N**	**N**	**N**	**ND**	**0.84**
**F30-A**	p.Y216C	c.647A>G	**N**	**M**	**8/15**	**classic**	**pos**	**Y**	**Y**	**Y**	**Y**	**N**	**N**	**Nor**	**N**	**N**	**N**	**ND**	**ND**
**F50-A**	p.C223R	c.667T>C	**N**	**M**	**7/29**	**classic**	**neg**	**Y**	**Y**	**Y**	**Y**	**N**	**Y**	**127.72**	**N**	**N**	**N**	**ND**	**4.33**
**F17-A**	p.F273L	c.817T>C	**N**	**M**	**7/30**	**classic**	**pos**	**N**	**Y**	**Y**	**Y**	**Y**	**Y**	**10.33**	**N**	**N**	**N**	**Y**	**0.12**
**F04-A**	p.A291T	c.871G>A	**N**	**M**	**17/28**	**classic**	**neg**	**Y**	**Y**	**Y**	**Y**	**ND**	**Y**	**6.41**	**Y**	**Y**	**N**	**Y**	**ND**
**F08-A**	p.A292P	c.874G>C	**N**	**M**	**5/14**	**classic**	**pos**	**N**	**Y**	**Y**	**Y**	**ND**	**N**	**Nor**	**N**	**Y**	**ND**	**ND**	**0.4**
**F62-A**	p.P293L	c.878C>T	**N**	**M**	**6/35**	**classic**	**pos**	**N**	**Y**	**Y**	**Y**	**Y**	**Y**	**61.88**	**N**	**N**	**Y**	**Y**	**0.29**
**F55-A**	p.M42I	c.126G>A	**Y**	**M**	**10/38**	**classic**	**pos**	**Y**	**N**	**Y**	**Y**	**Y**	**Y**	**48.15**	**N**	**Y**	**ND**	**Y**	**0.5**
**F55-B**	p.M42I	c.126G>A	**Y**	**F**	**7/23**	**classic**	**pos**	**N**	**Y**	**Y**	**Y**	**N**	**N**	**123.17**	**N**	**N**	**N**	**ND**	**6.7**
**F20-A**	p.D165Y	c.493G>T	**Y**	**M**	**5/19**	**classic**	**pos**	**N**	**Y**	**Y**	**Y**	**N**	**Y**	**98.97**	**N**	**N**	**N**	**ND**	**0.19**
**F11-A**	p.L206P	c.617T>C	**Y**	**M**	**8/29**	**classic**	**pos**	**Y**	**Y**	**Y**	**Y**	**N**	**Y**	**124.48**	**N**	**Y**	**Y**	**ND**	**0.75**
**F42-A**	p.E341G	c.1022A>G	**Y**	**M**	**6/17**	**classic**	**pos**	**Y**	**Y**	**N**	**Y**	**N**	**N**	**138.04**	**N**	**N**	**N**	**ND**	**3.85**
**F07-A**	p.R356G	c.1066C>G	**Y**	**M**	**9/17**	**classic**	**pos**	**Y**	**Y**	**Y**	**ND**	**ND**	**N**	**128.49**	**N**	**N**	**ND**	**ND**	**ND**
	Nonsense																		
**F35-A**	p.W81X	c.242G>A	**N**	**M**	**2/22**	**classic**	**pos**	**N**	**Y**	**N**	**N**	**Y**	**Y**	**137.40**	**N**	**N**	**N**	**Y**	**2.4**
**F35-B**	p.W81X	c.242G>A	**N**	**M**	**8/34**	**classic**	**pos**	**N**	**Y**	**Y**	**Y**	**Y**	**Y**	**75.56**	**N**	**N**	**Y**	**Y**	**1.9**
**F16-A**	p.W236X	c.708_709GA>AT	**N**	**M**	**5/31**	**classic**	**pos**	**N**	**Y**	**Y**	**ND**	**ND**	**Y**	**21.38**	**ND**	**N**	**N**	**ND**	**0.11**
**F49-A**	p.Q306X	c.916C>T	**N**	**M**	**12/23**	**classic**	**pos**	**Y**	**Y**	**Y**	**Y**	**Y**	**N**	**93.86**	**Y**	**N**	**Y**	**ND**	**1.33**
**F13-A**	p.R342X	c.1024C>T	**N**	**M**	**7/23**	**classic**	**pos**	**Y**	**Y**	**Y**	**Y**	**Y**	**Y**	**120.24**	**N**	**N**	**Y**	**Y**	**0.72**
	Splicings																		
**F10-A**		c.195-1G≥A	**N**	**M**	**8/20**	**classic**	**neg**	**Y**	**Y**	**Y**	**Y**	**N**	**Y**	**165.31**	**N**	**N**	**N**	**Y**	**ND**
	Deletions																		
**F28-A**	p.I239fsX249	c.718_719del AA	**N**	**M**	**5/22**	**classic**	**pos**	**Y**	**Y**	**Y**	**Y**	**Y**	**Y**	**127.63**	**N**	**N**	**N**	**ND**	**0.5**
**F23-A**	360Del Gly	c.1078_1080delGGT	**N**	**M**	**7/27**	**classic**	**pos**	**Y**	**Y**	**Y**	**Y**	**Y**	**Y**	**126.24**	**Y**	**N**	**N**	**ND**	**0.44**
**F39-A**	360Del Gly	c.1078_1080delGGT	**N**	**M**	**13/19**	**classic**	**pos**	**N**	**Y**	**Y**	**Y**	**N**	**Y**	**33.02**	**N**	**Y**	**N**	**Y**	**3.33**
**F77-A**	p.R11fsX120	c.32delG	**Y**	**M**	**6/20**	**classic**	**pos**	**Y**	**Y**	**Y**	**Y**	**Y**	**Y**	**131.78**	**N**	**N**	**N**	**Y**	**0**
**F63-A**	p.A20fsX30	c.59_72DelCCCTCGTTTCCTGG	**Y**	**M**	**10/13**	**classic**	**pos**	**Y**	**Y**	**Y**	**Y**	**N**	**N**	**151.26**	**N**	**N**	**N**	**ND**	**0.27**
**F26-A**	p.R193fsX240	c.579delG	**Y**	**M**	**5/13**	**classic**	**pos**	**N**	**Y**	**Y**	**Y**	**N**	**N**	**Nor**	**N**	**Y**	**N**	**ND**	**0.63**
**F26-B**	p.R193fsX240	c.579delG	**Y**	**F**	**18/35**	**classic**	**pos**	**N**	**Y**	**Y**	**Y**	**N**	**N**	**118.58**	**N**	**N**	**N**	**ND**	**ND**
**F65-A**		c.996_999delACAG	**Y**	**F**	**5/27**	**classic**	**pos**	**N**	**Y**	**N**	**Y**	**ND**	**Y**	**125.43**	**N**	**N**	**ND**	**Y**	**26.38**
**F21-A**	p.I359fsX379	c.1077_1120del44 bp	**Y**	**M**	**5/23**	**classic**	**pos**	**Y**	**Y**	**Y**	**Y**	**N**	**Y**	**129.83**	**Y**	**N**	**N**	**Y**	**0.21**
**F03-A**	p.G361fsX391	c.1082delG	**Y**	**F**	**10/32**	**classic**	**pos**	**Y**	**Y**	**Y**	**Y**	**N**	**Y**	**121.10**	**N**	**N**	**N**	**Y**	**12**
**F03-E**	p.G361fsX391	c.1082delG	**Y**	**F**	**12/35**	**classic**	**pos**	**N**	**Y**	**N**	**Y**	**N**	**Y**	**Nor**	**Y**	**N**	**N**	**ND**	**13.9**
**F03-F**	p.G361fsX391	c.1082delG	**Y**	**F**	**11/61**	**classic**	**pos**	**N**	**Y**	**Y**	**Y**	**Y**	**N**	**97.01**	**Y**	**N**	**Y**	**ND**	**31**
**F64-A**	p.T366fsX391	c.1098DelC	**Y**	**M**	**1/33**	**classic**	**pos**	**Y**	**Y**	**Y**	**Y**	**Y**	**Y**	**62.18**	**Y**	**N**	**Y**	**Y**	**0.12**
**F64-B**	p.T366fsX391	c.1098DelC	**Y**	**M**	**5/38**	**classic**	**pos**	**N**	**Y**	**Y**	**Y**	**Y**	**Y**	**11.75**	**Y**	**Y**	**Y**	**ND**	**0**
**F64-E**	p.T366fsX391	c.1098DelC	**Y**	**M**	**10/59**	**classic**	**pos**	**N**	**Y**	**Y**	**Y**	**Y**	**Y**	**40.60**	**N**	**Y**	**Y**	**ND**	**0.25**
**F64-F**	p.T366fsX391	c.1098DelC	**Y**	**F**	**8/24**	**classic**	**pos**	**N**	**Y**	**N**	**Y**	**N**	**Y**	**128.10**	**N**	**N**	**N**	**ND**	**13.56**
	Complex rearrangement																		
**F44-A**		c.793_809delins28bp	**Y**	**M**	**12/30**	**classic**	**pos**	**Y**	**Y**	**Y**	**Y**	**N**	**Y**	**6.85**	**Y**	**Y**	**N**	**Y**	**2.83**

Abbreviation: F:female; M:male; pos: positive; neg:negative; N:no; Y:yes; atypical-r: atypical renal-dominant phenotype; atypical-c: atypical cardiac-dominant phenotype; Nor: normal; ESRD: end-stage renal disease; LVH: left ventricular hypertrophy; ND: no detection.

$ Ocular manifestations included corneal opacity and/or tortuosity of the conjunctival and retinal vessels

& Cerebrovascular abnormalities included various clinical symptoms and signs, for example headache, vertigo/dizziness, transient ischemic attacks, ischemic strokes and/or cerebrovascular abnormalities found on MRI or CT scan.

^ Patient F06-A underwent nerve biopsy.

**Table 2:** Mutations and major clinic data of patients with renal-dominant phenotype.

	Mutations	Nucleotide change	Novel	Gender	Age at onset/ diagnosis	Clinical phenotypes	Family history	Angiokeratoma	Acroparesthesias	Hypohidrosis	Ocularchanges^$^	Hearing loss	Proteinuria	Baseline eGFR(EPI)	LVH	Hypertension	Cerebrovascular abnormalities^&^	Myeloid body	α-gal A activity(nmol/ml/h/mg)
	**Missense**																		
**F31-A**	p.G35R	c.103G>C	**N**	**M**	**34/42**	**atypical-r**	**pos**	**N**	**Y**	**N**	**ND**	**ND**	**Y**	ESRD*	**ND**	**Y**	**N**	**ND**	**0.27**
**F46-A**	p.A37T	c.109G>A	**N**	**M**	**34/34**	**atypical-r**	**pos**	**N**	**N**	**N**	**Y**	**Y**	**Y**	**73.14**	**N**	**N**	**N**	**Y**	**3.73**
**F05-A**	p.I91T	c.272T>C	**N**	**M**	**28/28**	**atypical-r**	**neg**	**N**	**N**	**Y**	**ND**	**ND**	**Y**	**62.08**	**N**	**Y**	**ND**	**Y**	**0.91**
**F53-A**	p.I91T	c.272T>C	**N**	**M**	**23/34**	**atypical-r**	**pos**	**N**	**N**	**N**	**Y**	**Y**	**Y**	**116.63**	**N**	**Y**	**Y**	**Y**	**0**
**F60-A**	p.I91T	c.272T>C	**N**	**M**	**33/34**	**atypical-r**	**pos**	**N**	**N**	**N**	**Y**	**N**	**Y**	**81.81**	**N**	**N**	**Y**	**Y**	**1.9**
**F60-B**	p.I91T	c.272T>C	**N**	**M**	**24/25**	**atypical-r**	**pos**	**N**	**N**	**N**	**Y**	**N**	**Y**	**135.97**	**N**	**N**	**N**	**Y**	**1.6**
**F67-A**	p.I91T	c.272T>C	**N**	**M**	**38/40**	**atypical-r**	**pos**	**N**	**N**	**N**	**Y**	**Y**	**Y**	**77.25**	**N**	**Y**	**Y**	**Y**	**0.62**
**F32-A**	p.R112H	c.335G>A	**N**	**M**	**19/19**	**atypical-r**	**neg**	**N**	**N**	**N**	**N**	**N**	**Y(NS)**	**129.59**	**Y**	**N**	**N**	**Y**	**7.46**
**F45-A**	p.R112H	c.335G>A	**N**	**M**	**33/33**	**atypical-r**	**pos**	**N**	**N**	**N**	**Y**	**N**	**Y**	**120.28**	**N**	**N**	**N**	**Y**	**2.71**
**F70-A**	p.R112H	c.335G>A	**N**	**M**	**35/37**	**atypical-r**	**neg**	**N**	**Y**	**N**	**Y**	**Y**	**Y**	**36.35**	**N**	**Y**	**N**	**Y**	**0.71**
**F71-A**	p.R112H	c.335G>A	**N**	**M**	**34/36**	**atypical-r**	**pos**	**N**	**N**	**N**	**Y**	**N**	**Y**	**41.92**	**N**	**Y**	**N**	**ND**	**0**
**F01-A**	p.C142R	c.424T>C	**N**	**F**	**40/45**	**atypical-r**	**neg**	**N**	**N**	**N**	**ND**	**ND**	**Y**	**83.39**	**N**	**Y**	**ND**	**Y**	**ND**
**F02-A**	p.R301Q	c.902G>A	**N**	**M**	**30/31**	**atypical-r**	**pos**	**Y**	**N**	**N**	**N**	**N**	**Y**	**89.84**	**Y**	**Y**	**N**	**Y**	**0.85**
**F14-A**	p.R301Q	c.902G>A	**N**	**M**	**36/48**	**atypical-r**	**pos**	**N**	**N**	**N**	**N**	**Y**	**Y**	**6.04**	**Y**	**Y**	**Y**	**ND**	**0.41**
**F15-A**	p.R301Q	c.902G>A	**N**	**M**	**34/34**	**atypical-r**	**pos**	**N**	**N**	**N**	**ND**	**N**	**Y**	**87.71**	**ND**	**N**	**N**	**Y**	**0.42**
**F09-A**	p.W349R	c.1045T>A	**N**	**F**	**44/45**	**atypical-r**	**pos**	**N**	**N**	**N**	**N**	**N**	**Y**	**72.72**	**Y**	**Y**	**N**	**Y**	**ND**
**F52-A**	p.Y88C	c.263A>G	**Y**	**M**	**28/33**	**atypical-r**	**pos**	**N**	**N**	**N**	**Y**	**ND**	**Y**	**127.63**	**Y**	**N**	**ND**	**Y**	**0.06**
**F73-A**	p.V124G	c.371T>G	**Y**	**M**	**30/30**	**atypical-r**	**pos**	**N**	**N**	**N**	**N**	**N**	**Y**	**85.14**	**Y**	**N**	**N**	**Y**	**0.63**
**F74-A**	p.V124G	c.371T>G	**Y**	**F**	**26/31**	**atypical-r**	**neg**	**N**	**N**	**N**	**Y**	**N**	**Y**	**127.14**	**N**	**N**	**N**	**Y**	**61**
**F59-A**	p.H125T	c.373C>T	**Y**	**M**	**31/41**	**atypical-r**	**pos**	**N**	**Y**	**N**	**Y**	**Y**	**Y**	**3.88**	**Y**	**Y**	**Y**	**ND**	**0.78**
**F72-A**	p.A160D	c.479C>A	**Y**	**M**	**51/59**	**atypical-r**	**pos**	**N**	**N**	**N**	**N**	**N**	**Y**	**46.81**	**Y**	**Y**	**N**	**Y**	**4.79**
**F57-A**	p.G163R	c.487G>C	**Y**	**F**	**45/47**	**atypical-r**	**pos**	**N**	**N**	**N**	**Y**	**N**	**Y**	**108.99**	**N**	**N**	**N**	**Y**	**77.73**
**F34-A**	p.R196T	c.587G>C	**Y**	**M**	**50/55**	**atypical-r**	**pos**	**N**	**N**	**N**	**ND**	**N**	**Y**	**47.08**	**N**	**Y**	**N**	**Y**	**0.72**
**F66-A**	p.P205S	c.613C>T	**Y**	**M**	**30/31**	**atypical-r**	**pos**	**N**	**N**	**N**	**N**	**N**	**Y**	**111.72**	**N**	**N**	**N**	**ND**	**0.75**
**F33-A**	p.M290V	c.868A>G	**Y**	**M**	**26/30**	**atypical-r**	**neg**	**N**	**N**	**N**	**N**	**N**	**Y**	**Nor**	**N**	**N**	**N**	**Y**	**ND**
**F54-A**	p.M296T	c.887T>C	**Y**	**M**	**49/49**	**atypical-r**	**pos**	**N**	**Y**	**N**	**ND**	**ND**	**Y**	**77.24**	**Y**	**N**	**N**	**Y**	**0**
**F29-A**	p.R356G	c.1066C>G	**Y**	**M**	**50/64**	**atypical-r**	**neg**	**N**	**N**	**N**	**N**	**Y**	**Y**	**69.52**	**N**	**Y**	**N**	**Y**	**0.84**
	Splicings																		
**F58-A**		c.194+2T>C	**N**	**F**	**28/34**	**atypical-r**	**pos**	**N**	**N**	**N**	**Y**	**N**	**Y**	**111.23**	**N**	**Y**	**ND**	**Y**	**33.13**

Abbreviation: F:female; M:male; pos: positive; neg:negative; N:no; Y:yes; atypical-r: atypical renal-dominant phenotype; atypical-c: atypical cardiac-dominant phenotype; Nor: normal; ESRD: end-stage renal disease; LVH: left ventricular hypertrophy; ND: no detection.

$ ocular manifestations included corneal opacity and/or tortuosity of the conjunctival and retinal vessels

& Cerebrovascular abnormalities included various clinical symptoms and signs, for example headache, vertigo/dizziness, transient ischemic attacks, ischemic strokes and/or cerebrovascular abnormalities found on MRI or CT scan.

* Patient F31-A was already undergoing persistent hemodialysis.

**Table 3:** Mutations and major clinic data of patients with cardiac-dominant and uncertain phenotypes.

	Mutations	Nucleotide change	Novel	Gender	Age at onset/ diagnosis	Clinicalphenotypes	Family history	Angiokeratoma	Acroparesthesias	Hypohidrosis	Ocularchanges^$^	Hearing loss	Proteinuria	Baseline eGFR(EPI)	LVH	Hypertension	Cerebrovascular abnormalities ^&^	Myeloid body	α-gal A activity(nmol/ml/h/mg)
	**Missense**																		
**F31-B**	p.G35R	c.103G>C	**N**	**F**	**20/20**	**atypical**	**Pos**	**N**	**N**	**N**	**Y**	**N**	**N**	**118.18**	**N**	**N**	**Y**	**ND**	**26.94**
**F06-B**	p.G132R	c.394G>C	**N**	**F**	**53/55**	**atypical-c**	**Pos**	**N**	**N**	**N**	**N**	**Y**	**N**	**92.42**	**Y**	**Y**	**ND**	**ND**	**13.6**
**F14-B**	p.R301Q	c.902G>A	**N**	**F**	**50/58**	**atypical-c**	**Pos**	**N**	**N**	**N**	**N**	**N**	**N**	**63.61**	**Y**	**Y**	**Y**	**ND**	**18.3**
**F14-C**	p.R301Q	c.902G>A	**N**	**F**	**25/57**	**atypical-c**	**Pos**	**N**	**N**	**N**	**N**	**Y**	**N**	**86.29**	**Y**	**N**	**Y**	**ND**	**59.5**
**F14-E**	p.R301Q	c.902G>A	**N**	**M**	**52/53**	**atypical-c**	**Pos**	**N**	**N**	**N**	**N**	**N**	**Y**	**95.72**	**Y**	**Y**	**Y**	**ND**	**4.6**
**F76-A**	p.V199A	c.596T>C	**Y**	**M**	**?/11**	**uncertain**	**Pos**	**N**	**N**	**N**	**Y**	**N**	**N**	**173.53**	**N**	**N**	**N**	**ND**	**23.19**
	Deletions																		
**F64-C**	p.T366fsX391	c.1098DelC	**Y**	**F**	**61/61**	**Atypical-c**	**Pos**	**N**	**N**	**N**	**Y**	**Y**	**N**	**98.78**	**N**	**Y**	**Y**	**ND**	**85.36**

Abbreviation: F:female; M:male; pos: positive; neg: negative; N:no; Y:yes; atypical-r: atypical renal-dominant phenotype; atypical-c: atypical cardiac-dominant phenotype; Nor: normal; ESRD: end-stage renal disease; LVH: left ventricular hypertrophy; ND: no detection.

$ Ocular manifestations included corneal opacity and/or tortuosity of the conjunctival and retinal vessels

& Cerebrovascular abnormalities included various clinical symptoms and signs, for example headache, vertigo/dizziness, transient ischemic attacks, ischemic strokes and/or cerebrovascular abnormalities found on MRI or CT scan.

Thirty-one missense mutations were identified in 48 patients (39 males, 9 females) belonging to 41 families. Fourteen male probands carrying the following mutations presented the classic FD: p.M42I, p.G132R, p.D165Y, p.G183D, p.T194I, p.L206P, p.Y216C, p.C223R, p.F273L, p.A291T, p.A292P, p.P293L, p.E341G and p.R356G. Twenty-two male patients with the following mutations had atypical renal-dominant FD: p.G35R, p.A37T, p.Y88C, p.I91T, p.R112H, p.V124G, p.H125T, p.A160D, p.R196T, p.P205S, p.M290V, p.M296T and p.R301Q. The male proband of F07 with classical manifestations differed from the male proband of F29 carrying the same mutation p.R356G but presenting as atypical renal-dominant phenotype. A male member from F14 carrying mutation p.R301Q presented atypical cardiac-dominant FD (unlike the proband with renal-dominant phenotype). An 11-year-old male patient with mutation p.V199A who was discovered by a family survey had no clinical presentations except for corneal opacities. Only one female patient carrying mutation p.M42I presented as a classical FD. Four female patients with mutations p.V124G, p.C142R, p.G163R and p.W349R had atypical renal-dominant FD. One female member from F06 carrying mutation p.G132R and two female members from F14 carrying mutation p.R301Q presented as atypical cardiac-dominant FD. A 20-year old female member, niece of proband F31, showed only corneal opacities and small infarcts in the frontal and parietal lobe on cerebral MRI examination.

All 17 male and six out of seven female patients carrying frameshift mutation presented classic phenotype. A female patient from F64 carrying deletion c.1098delC had atypical cardiac-dominant FD. A female proband with a reported splicing mutation (c.194+2T>C) had the atypical renal-dominant FD.

### Genotype, phenotype and α-gal A enzyme activity

**Fig 2:**
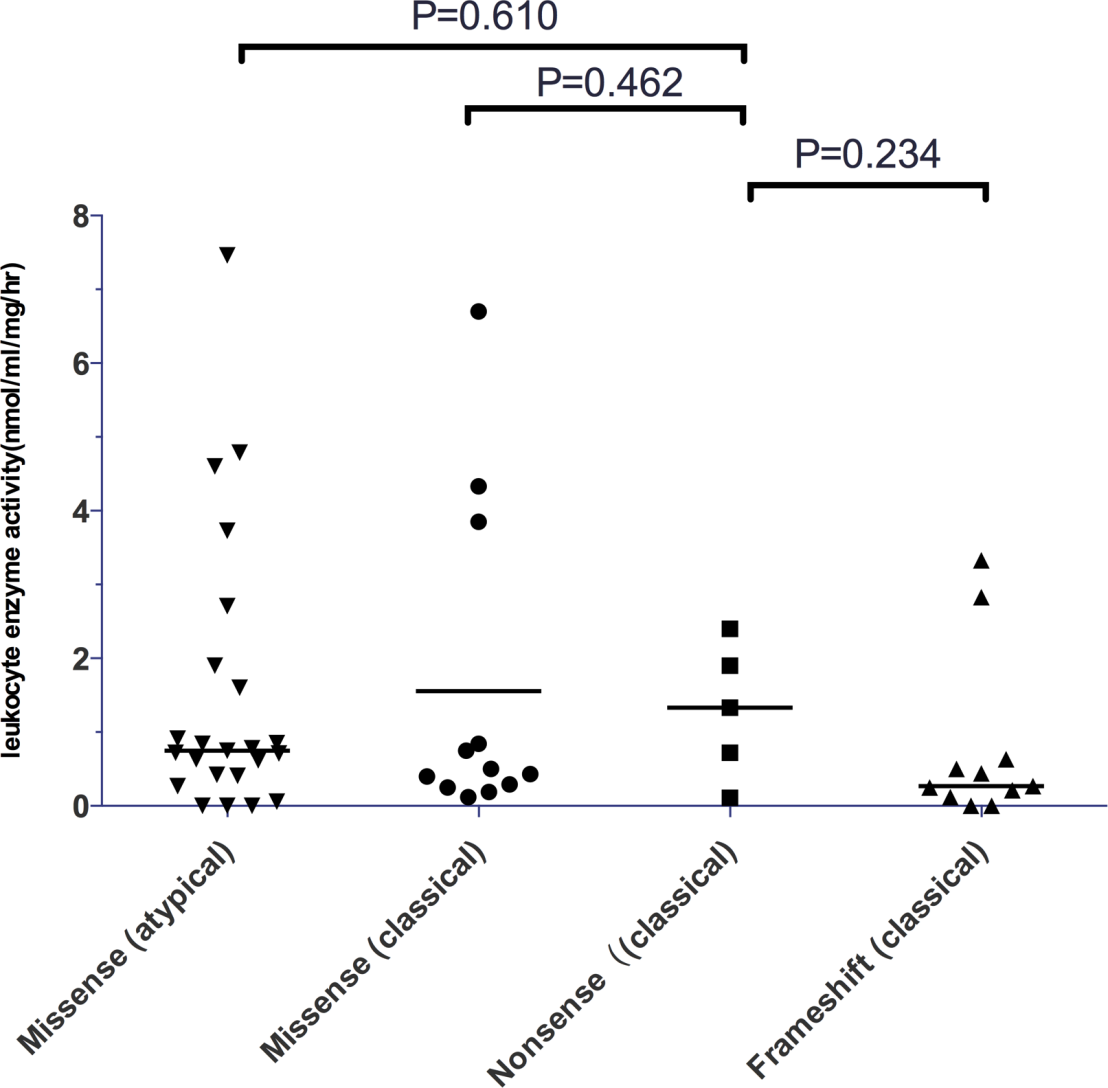
The relationship between genotype, clinical phenotypes and α-galactosidase A activity in male patients.

**Fig 3:**
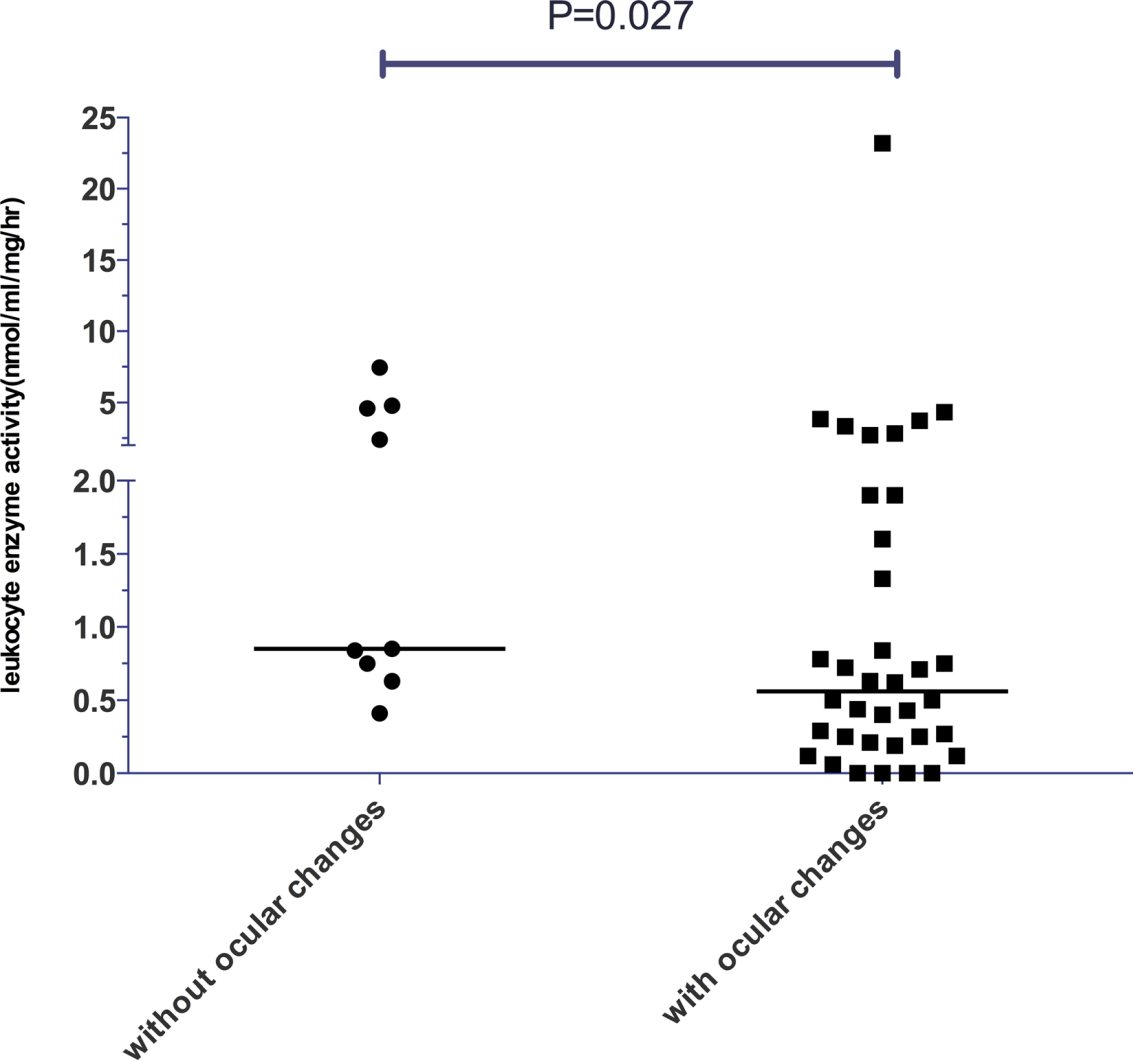
The relationship between the level of α-galactosidase A activity and ocular changes in male patients.

α-gal A enzyme activity was detected in 65 patients (51 male and 14 female.) from 51 families. All of the male patients had extremely low enzyme activity (Tables 1–3). The α-gal A activity was 0.44 nmol/ml/h/mg (0~4.33 nmol/ml/h/mg) for male patients with classical FD as compared to 0.75 nmol/ml/h/mg (0~7.46 nmol/ml/h/mg) for male patients with atypical FD. No statistically significant difference was found between these two groups. Among 27 male patients with classical phenotype, 11 carried missense mutations, 5 carried nonsense and 11 carried frameshift mutations. All male patients with atypical phenotypes carried missense mutations. Comparison of the enzyme activity among patients with different types of mutations and clinical phenotypes revealed no significant difference (Fig 2). The residual enzyme activity for male patients with ocular manifestations was significantly lower than that for patients without ocular manifestations (*P* = 0.027; Fig 3). No significant variation was found between patients with and without neurological pain, angiokeratoma, hearing loss, hypertension or LVH (*P*≥0.05).

**Fig 4:**
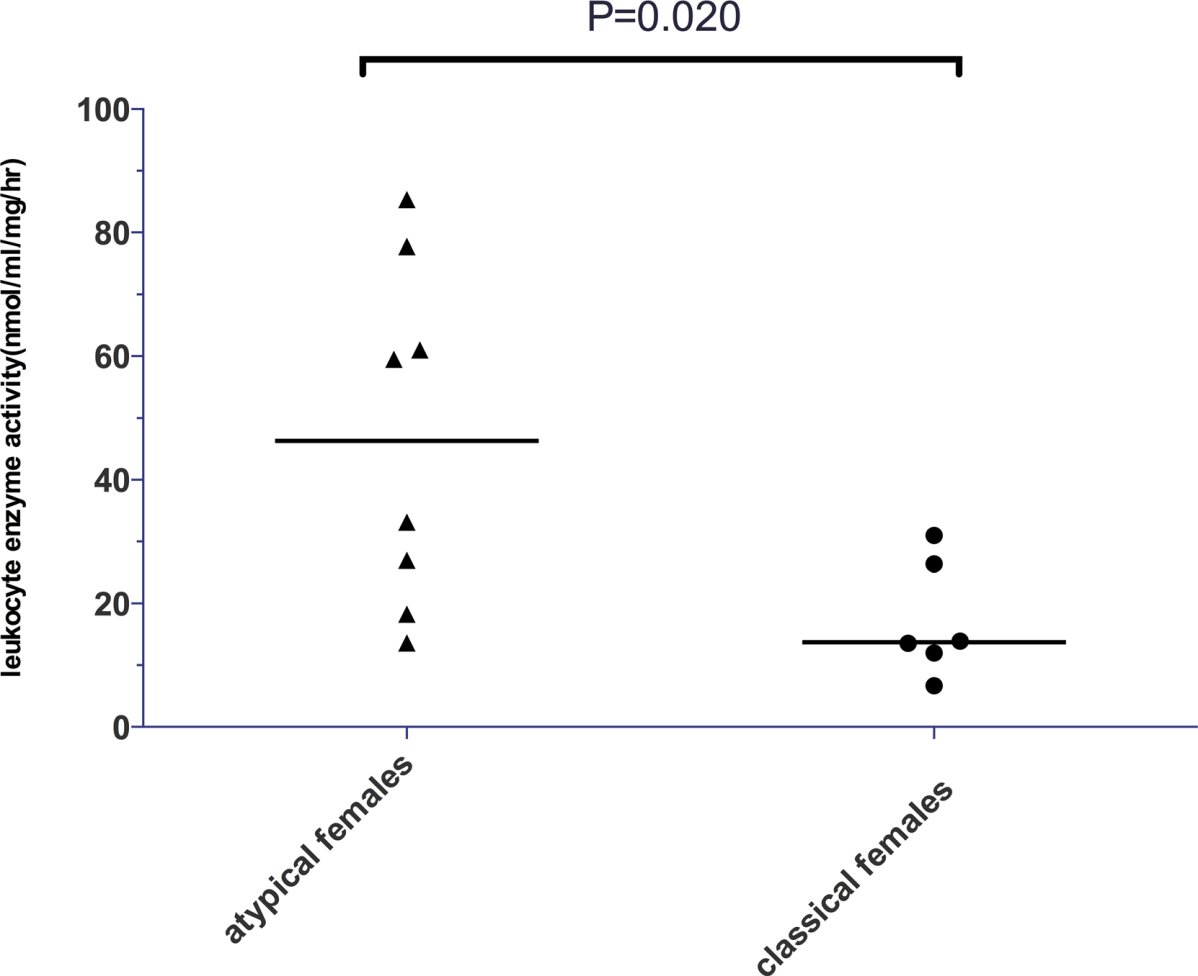
Comparison of the level of α-galactosidase A activity in female patients with classical FD and atypical FD.

Reduced α-gal A activity was observed in all female patients with classical FD and half of female patients with atypical FD, totally 71.4% of female patients. The α-gal A activity level for female patients with classical FD was 13.73 nmol/ml/h/mg (6.7~31.0nmol/ml/h/mg), which was significantly lower than that for female patients with atypical phenotype (46.32 nmol/ml/h/mg, 13.6~85.36nmol/ml/h/mg) (*P* = 0.020; Fig 4). Among six female patients with classical FD, five carried deletion mutations and one carried a missense mutation (p.M42I). Six out of eight female patients with atypical FD possessed missense mutations and one deletion, one splicing mutation (Tables 1–3).

## Discussion

In our study, we found 47 mutations which were located in all seven exons of gene GLA. Most of the mutations were small nucleotide variations including missense, nonsense, small deletions and splicing mutations. Large deletion and complex rearrangement accounted for only 6.4% of cases, similar to the findings from other studies [16–19].

This study identified 24 previously reported mutations and 23 novel mutations. The three-dimensional structure of human α-gal A determined by x-ray crystallography revealed α-gal A as a homodimeric glycoprotein with each monomer containing two domains: the N-terminal domain (residues 32–328) and the C-terminal domain (residues 329–421) [20]. The N-terminal domain contains the active site, which consists of side chain residues of W47, D92, D93, Y134, C142, K168, D170, C172, E203, L206, Y207, R227, D231, D266, and M267. To date, more than 600 GLA gene mutations have been reported. Although changes of all residues in the active site have been found, most mutations are not located at the active site [21]. Our study confirmed this finding. Frameshift and complex rearrangement mutations might result in a failure to synthesize complete α-gal A. In our study, these were observed in one novel complex rearrangement, seven novel frameshift mutations and one reported splicing mutation. Missense mutations were the most frequent mutation type, accounting for 66.0% of mutations in our study. Such mutations might induce impaired kinetic and/or stability properties of the enzyme, resulting in decreased enzymatic activity or increased susceptibility to photolytic attack. Lin *et al* [22] indicated that the intronic splicing mutation IVS4+919G>A was commonly found in Taiwan Chinese FD patients with cardiac-dominant phenotype. Unfortunately, our gene test mainly focused on exons and flanking intronic regions, while the rest intronic regions including position IVS4+919G were not sequenced.

While a great number of gene mutations have been identified in FD patients, few studies have been reported on genotype-phenotype correlations [12, 23]. In this study, we first analysed the effects of genotype on clinical phenotype in FD patients. We found that classic phenotype was present in all male FD patients with frameshift mutations. Male patients from unrelated families carrying the same mutations had similar clinical manifestations. Similar findings were reported in male patients with mutations W81X [24, 25], p.W236X [26], p.Q306X [11], p.R342X [27–29] and c.718_719del AA [30–32].

The situation is complicated in male patients with missense mutations. Four novel missense mutations (p.M42I, p.D165Y, p.L206P, and p.E341G) and nine reported missense mutations (p.G132R, p.G183D, p.T194I, p.Y216C, p.C223R, p.F273L, p.A291T, p.A292P, p.P293L) resulted in the classical phenotype. Among them, only one novel mutation was located in the active site (p.L206P). Different nucleotide substitutions, in position M42 (M42T, M42V), D165 (D165H, D165V), G183 (G183S, G183R), C223 (C223Y), P293 (P293T) and E341 (E341D, E341K), have been reported by other groups who also reported the classic phenotype [11, 12, 16, 18, 33–35]. These results indicate that these positions are very important to the conformation and function of α-gal A. Eight novel missense mutations p.Y88C, p.V124G, p.H125T, p.A160D, p.R196T, p.P205S, p.M290V, p.M296T and 2 known mutations p.G35R and p.A37T resulted in the atypical renal-dominant phenotype. Unlike our findings, patients carrying previously reported mutations p.M290I and p.P205R, p.P205T [11, 12, 25, 30] presented the classic phenotype, and patients carrying the mutations p.M296V, p.M296I, p.M296L [17, 22, 31] had the atypical cardiac-dominant FD. Different amino acids have different structures and features, which might result in different protein conformation or characteristics.

We found male patients with the same mutation presented different clinical manifestations. For instance, two male probands carrying the same novel mutation (p.R356G), one presented the classic phenotype and the other the renal-dominant phenotype. This phenomenon was also observed in mutations p.I91T, p.R112H and p.R301Q. All male patients with mutation p.I91T and p.R112H in our group presented atypical renal-dominant FD, whereas atypical cardiac-dominant phenotype have been reported in such patients by other researchers [19, 23]. Not only classical FD, but also the atypical FD including renal-dominant or cardiac-dominant phenotypes, were reported in patients carrying p.R301Q from different families [26, 29]. However, to our knowledge, phenotypic variation in hemizygote males from the same family has not been described before. R112, R301, R356 were all located at a CpG dinucleotide. The same nucleotide substitution in a CpG position might result in different phenotypes, but this mechanism needs to be elucidated. The reason for the phenotypic variation among male patients with mutation p.I91T is still not clear. These findings suggest that besides nucleotide substitution, other genetic or non-genetic factors might be associated with the clinical phenotype.

Marked deficiency of α-gal A activity is a definitive symptom for the diagnosis of hemizygote male patients with FD [36]. However, the residual enzyme activity has no obvious association with genotype and phenotype for male patients. We further analysed the association of residual enzyme activity with clinical manifestations, and a statistically significant relationship between the level of enzyme activity and ophthalmologic changes was found. This differed from the results reported by Altarescu *et al* [23] who found significant association between enzyme activity level and neurologic pain.

For female patients, genotype-phenotype correlation was more complicated. We found that female patients with frameshift mutations were more likely to had the classical clinical manifestations and those with missense mutations were inclined to present atypical manifestations. Compared with male patients, female patients with the same mutations usually presented less severe clinical manifestations. In fact, clinical presentations in female patients were more variable ranging from being asymptomatic to, occasionally, being as severely affected as male patients while males were usually severely affected (Tables 1–3). The underlying mechanism of developing symptoms in heterozygous females is still unknown–most have almost normal enzyme activity and X-chromosome inactivation (XCI) takes place in each cell at the beginning of embryogenesis. The random process of XCI means that their tissues should be a mosaic of normal and deficient cells [37, 38]. XCI was reported to be a major factor determining the clinical severity in female heterozygotes [32]. Recently, Germain DP *et al*. [7] also indicated that the existence of skewed XCI significantly impacts the phenotype and natural history of FD in females. For example, significant differences in residual α-Gal levels depending on the direction and degree of skewing of XCI were evidenced in his research. In our study, reduced α-gal A activity was found in all female patients with classical FD. The level of α-gal A activity was significantly lower for female patients with the classical FD compared with female patients with the atypical phenotypes. In accordance with previous studies, the diverse level of enzyme activity found in heterozygote women presumably results from skewing of XCI. This result also suggests that the enzyme activity level might be associated with the clinical severity of female patients.

Although this is a large Chinese Fabry study with very detailed genetic and clinical information, genotype/phenotype correlations in FD patients still need to be fully established since most patients have unique mutations. Collecting more extensive clinical information from patients with the same genotype through further family investigation is needed to verify our findings. Phenotypic variation in male patients with same mutation warrants further investigation.

## Supporting Information

S1 Fig **Ultrastructural findings in renal tissue from a male patient with classical Fabry Disease** (A. myeloid bodies clustered in podocyte; B. myeloid bodies seen in mesangial and endothelial cells; C. myeloid bodies seen in renal tubular epithelial cells).
(TIFF)

S1 Table Diagnostic criteria for a definite classification of phenotype of Fabry Disease. (DOCX)
