# Supplementary figures and images for: Genotype: A Crucial but Not Unique Factor Affecting the Clinical Phenotypes in Fabry Disease
Source: PLoS One. 2016 Aug 25;11(8):e0161330. doi: 10.1371/journal.pone.0161330 (PMC4999276; doi:10.1371/journal.pone.0161330)

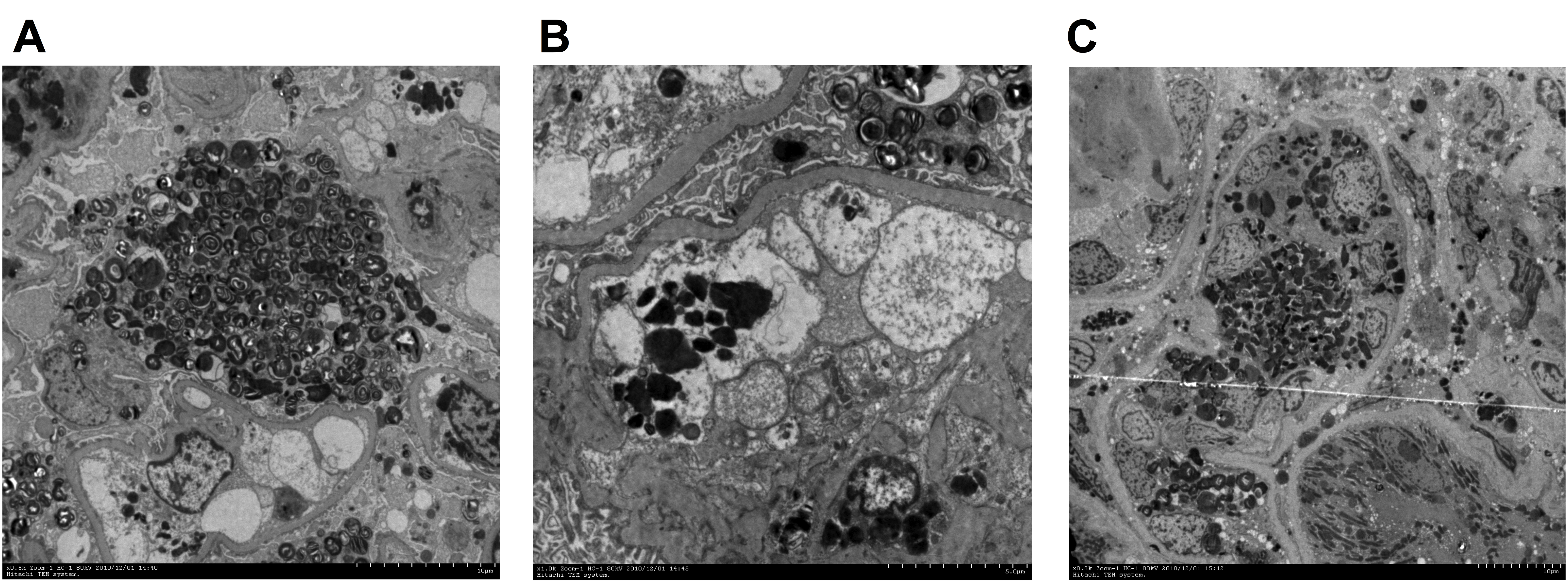

Supplement: S1 Fig — Ultrastructural findings in renal tissue from a male patient with classical Fabry Disease (A. myeloid bodies clustered in podocyte; B. myeloid bodies seen in mesangial and endothelial cells; C. myeloid bodies seen in renal tubular epithelial cells). (TIFF) [file pone.0161330.s001.tiff]
