# Supplementary material for: Genotype: A Crucial but Not Unique Factor Affecting the Clinical Phenotypes in Fabry Disease
Source: PLoS One. 2016 Aug 25;11(8):e0161330. doi: 10.1371/journal.pone.0161330 (PMC4999276; doi:10.1371/journal.pone.0161330)
Supplement: S1 Table — (DOCX) [file pone.0161330.s002.docx]

Supplement Table1 Diagnostic Criteria for a Definite Classification of Phenotype of Fabry Disease

| Definite classification of phenotype | | | |
| --- | --- | --- | --- |
| Classical | | Atypical | |
| Males  A  GLA mutation/variant | Females  A  GLA mutation/variant | Males  A  GLA mutation/variant | Females  A  GLA mutation/variant |
| B  α-GalA deficiency of ≤5% of reference value in leukocytes | B  Deficient α-GalA in leukocytes | B  Deficient α-GalA in leukocytes | B  Normal or deficient α-GalA in leukocytes |
| C  Onset age was less than 25 years | | C  Myeloid bodies in tissue cells using EM | |
| D1 ≥1 characteristic FD sign/symptom (Fabry neuropathic pain, cornea verticillata or clustered angiokeratoma)  D2 ≥2 characteristic FD sign/symptom (Fabry neuropathic pain, cornea verticillata or clustered angiokeratoma) | | D  An FD-like sign or symptom that is not specific for FD | |
| E  A family member with a definite diagnosis of classical FD | | E Predominantly renal involvement | |
|  | | F Predominantly cardiac involvement | |
| A+B+C+D1 or A+B+C+E  A+C+D2 or A+C+D1+E | | A+B+D+E or A+C+D+E(renal-dominant)  A+B+D+F or A+C+D+F (cardiac-dominant) | |

*Definitions of special FD symptom

1. Fabry neuropathic pain meets the ‘characteristic clinical criteria’ if there is neuropathic pain in hands and/or feet, starting before age 18 years or increasing with heat, fever. There is no other cause for neuropathic pain.
2. Angiokeratoma meet the ‘characteristic clinical criteria’ if they are clustered and present in characteristic areas: bathing trunk area, lips, and umbilicus. There is no other cause for angiokeratoma.
3. Cornea verticillata meets the ‘characteristic clinical criteria’ if there is a whorl like pattern of corneal opacities. There is no other cause (medication induced, among other: amiodarone, chloroquine).
